# Retraction Note: Experimental and TDDFT materials simulation of thermal characteristics and entropy optimized of Williamson Cu-methanol and Al_2_O_3_-methanol nanofluid flowing through solar collector

**DOI:** 10.1038/s41598-025-93134-x

**Published:** 2025-03-12

**Authors:** Wasim Jamshed, Mohamed R. Eid, Ahmed F. Al-Hossainy, Zehba Raizah, El Sayed M. Tag El Din, Tanveer Sajid

**Affiliations:** 1 Department of Mathematics, Capital University of Science and Technology (CUST), Islamabad, 44000 Pakistan; 2 Department of Mathematics, Faculty of Science, New Valley University, Al-Kharga, 72511 Al-Wadi Al-Gadid Egypt; 3 Department of Mathematics, Faculty of Science, Northern Border University, 1321 Arar, Saudi Arabia; 4 Department of Chemistry, Faculty of Science, New Valley University, Al-Kharga, 72511 Al-Wadi Al-Gadid Egypt; 5 Department of Mathematics, College of Science, King Khalid University, Abha, Saudi Arabia; 6 Electrical Engineering, Faculty of Engineering and Technology, Future University in Egypt, New Cairo, 11835 Egypt

Retraction of: *Scientific Reports* 10.1038/s41598-022-23025-y, published online 28 October 2022

The Editors have retracted this Article. After publication, concerns were raised regarding repetitive patterns in the top left corner of Fig. 3a, and highly similar particles and unusual features in Fig. 3b.

The Authors have not provided the underlying raw data files for validation upon request.

The Editors therefore no longer have confidence in the presented data.

Ahmed F. Al-Hossainy does not agree to this retraction. None of the other Authors have responded to any correspondence from the Publisher about this retraction.

